# Stress in ICU Caregivers: Does it Lie in the Eyes of the Beholder?

**DOI:** 10.5005/jp-journals-10071-23159

**Published:** 2019-05

**Authors:** Deven Juneja, Anish Gupta, Atul P Kulkarni

**Affiliations:** 1,2 Institute of Critical Care Medicine, Max Super Speciality Hospital, Saket, New Delhi, India; 3 Division of Critical Care Medicine, Department of Anesthesiology, Critical Care and Pain, Tata Memorial Hospital, Homi Bhabha National Institute, Mumbai, Maharashtra, India

## Abstract

**How to cite this article:** Juneja D, Gupta A, Kulkarni AP. Stress in ICU Caregivers: Does it Lie in the Eyes of the Beholder? Indian J Crit Care Med 2019;23(5):203–204.

Stress can be defined as a physical, mental or emotional factor which leads to bodily or mental tension. Stress may arise when an individual is unable to cope with a particular situation or circumstance. A person's ability to deal with difficult situations differs, with some people handling these situations in a better way than others. A concept to understand the person's ability to cope up with stress is the “stress jug”. This concept explains the individual's capacity to absorb stress: larger the jug, better the ability. However, no matter how big the jug is, a point will come when it is full and beyond that it would not be able to take any more stress. Hence, at this point, even a trivial increase in stress may lead to a seemingly disproportionate stress response.

The environment in the intensive care unit (ICU) can be highly stressful, especially for the patients and their relatives. Only after 1990, it was realized that the family members of the ICU patients could potentially develop psychological conditions, when one of the earlier studies showed that more than 50% of family members of critically ill traumatic brain injury (TBI) patients developed symptoms of anxiety, depression, hypochondriasis, and suicidal tendency.^1^

Caregivers of critically ill patients may develop symptoms related to stress, anxiety, depression, and complicated grief. These clusters of symptoms have been classically identified as “post-intensive care syndrome – family”.^2^ In a large multi-center French trial, conducted in 43 ICUs and including 836 family members of critically ill patients, a great majority (72.7%) of family members reported symptoms of anxiety or depression.^3^ The incidence was even higher, up to 84%, among the spouses of these patients.^3^ In other studies, the reported prevalence of depression in family members of critically ill patients has been reported to be in the range of 15 to 35%, whereas the reported prevalence rate of anxiety is as high as 35 to 73%.^4^

In extreme cases, these symptoms may last even up to 4 years after ICU discharge or death of the patient. Stress for prolonged period may lead to destructive changes in the body causing serious problems like depression, cardiac diseases, hypertension, stroke, indigestion, ulcers, headache, and body aches.^5^

Apart from having clinical and psychological effects on the well-being of these caregivers, these symptoms may also affect their ability to care and take rational decisions for their critically ill patients.^6^

Several factors have been identified which may contribute to increased levels of stress, depression, and anxiety. These include age and gender of the caregiver, relationship with the patient, type of ICU the patient is admitted in, and the educational status of the family. In addition, inconsistent information and irregular family meetings with the clinicians have also been shown to increase stress and anxiety of the caregivers.^3,4^

As the physicians are classically trained to concentrate on the care of critically ill patients, the requirements of their caregivers may get ignored. Hence, it becomes imperative to include concern for caregivers of these critically ill patients as a part of their holistic care. To this effect, clinical practice guidelines^7^ and a consensus document^8^ have been published which incorporate a family-centered approach for managing critically ill patients in ICU. These recommendations suggest assessment of psychological symptoms of the caregivers and to analyse their stress and anxiety levels. This approach will be beneficial in improving their satisfaction levels and reduce occurrence of these psychological symptoms.

However, before these recommendations can be incorporated into our routine critical care, more understanding of these symptoms and their implications is needed. Moreover, social and cultural factors differ in different regions of the world and these factors influence how people perceive and cope up with stress. Hence, it is essential to perform studies especially pertaining to the Indian scenario.

In this issue, Kanmani and colleagues have presented their data from caregivers of 60 patients admitted in ICU with TBI.^9^ In their descriptive study, they reported that most of the caregivers were young males, who were manual labourers by profession. The caregivers of these critically ill patients experienced severe financial burden, higher levels of family burden, severe psychological distress, and moderate levels of anxiety. The authors also reported that perception of stress differed among male and female caregivers, with females coping up better with stress. In addition, severity of TBI was directly related to the severity of caregiver burden.^9^

This study highlights the fact that the physical and mental health issue of the caregivers of critically ill patients is a significant but an often neglected issue. Patients with trauma and TBI form a special group as they are generally younger, do not have many comorbidities and their ICU admission is sudden and unexpected. Hence, the caregivers are not mentally and financially prepared for providing such care, which may add to their stress and financial burden. Patients with severe TBI are particularly vulnerable, and they may require prolonged ICU and hospital care adding to the stress of their caregivers. This study also stresses upon the importance of recognizing and addressing the issue of stress among caregivers of critically ill patients and adopting a more family-oriented critical care practice.

As the relatives of the critically ill patients play an important part in their ICU care and are expected to make unprecedented decisions regarding their management and deal with social and economic difficulties, they are under tremendous stress. They may develop psychological symptoms, which not only affects their general well-being but also makes them less efficient in taking decisions regarding their patients. Early recognition and proper addressal of these issues should form an integral part of holistic care of critically ill patients.
